# Using pre-existing social networks to determine the burden of disease and real-life needs in rare diseases: the example of Thygeson's superficial punctate keratitis

**DOI:** 10.1186/s13023-021-01707-6

**Published:** 2021-01-30

**Authors:** Rana Saad, Sami Saad, Oscar Haigh, Domitille Molinari, Marc Labetoulle, Antoine Rousseau

**Affiliations:** 1 Service d’Ophtalmologie, Assistance Publique-Hôpitaux de Paris, Hôpital Bicêtre, Université Paris-Saclay, Centre de Référence Maladies Rares en Ophtalmologie (OPHTARA), 78, rue du Général Leclerc, 94275 Le Kremlin Bicêtre, France; 2 Centre Hospitalier National d’Ophtalmologie des Quinze-Vingts, Paris, France; 3 CEA, Center for Immunology of Viral, Auto-immune, Hematological and Bacterial diseases (IMVA-HB/IDMIT), Fontenay-aux-Roses, Le Kremlin-Bicêtre, France; 4 Unité de Recherche Clinique Paris-Sud, Hôpital Bicêtre, Le Kremlin Bicêtre, France

**Keywords:** Thygeson’s superficial punctate keratitis, Social network, Clinical journey, Quality of life, Burden of disease

## Abstract

**Background:**

Thygeson’s superficial punctate keratitis (TSPK) is a rare and still poorly understood disease of the ocular surface, responsible for recurrent episodes of photophobia and eye pain. While TSPK is considered as a benign condition, a subset of patients has frequent recurrences or even chronic disease, two situations in which there are currently no therapeutic guidelines. We used a preexisting Facebook TSPK patient support group to assess the clinical journey and the burden of disease of TSPK.

**Results:**

An online survey was sent to the patient support group. The first part of the questionnaire gathered information on demographics and the patient’s clinical journey [diagnostic modalities, symptoms, duration and frequency of recurrent episodes (RE), efficacy and tolerance to treatments]. The second part focused on quality of life (QoL) using the Ocular Surface Disease-QoL (OSD-QoL) questionnaire. Seventy-two patients out of 595 members of the support group completed the questionnaire during the 3-months study period. Eighty percent of patients developed symptoms before 30 years old, and 47% reported a delay in the diagnosis above 1 year. Sixty percent of patients reported over 5 RE yearly, and 18% of RE lasted more than 3 months. Forty percent of all patients used cyclosporine eyedrops (50% of those with > 5 episodes/year) and it was perceived as effective by 72% of these patients. The impact on daily life activities was judged as severe by 22% of patients, while 38% reported reduced professional activity and 80% were deeply saddened by their eye condition.

**Conclusion:**

TSPK patients may present with frequent recurrences and/or chronic disease, that result in a severe impact on QoL, and an off-label use of topical immunomodulatory eye drops, suggesting the urgent need for controlled studies. The utility of using social networks for rare ophthalmic disease research includes, faster data collection, data from patients across the globe, and also raises relevant questions about their real needs.

## Introduction

Thygeson’s superficial punctate keratitis (TSPK) is a rare ocular surface disease, characterized by recurrent episodes of photophobia and foreign body sensation, associated with multiple white–grey superficial epithelial lesions, without stromal involvement and mild or absent conjunctival hyperemia [1–3]. The etiology remains unclear, although both viral and immunologic mechanisms have been implicated [1, 4–7]. As TSPK is a rare disease, limited data are available on the epidemiology and natural history of this disease. Hence, the prevalence and distribution of the disease could be underestimated, as there is a dearth of recent publications.

TSPK seems to affect more females than males, age of onset is usually in the third decade of life, but can affect people of all ages, from 2 years old to 71 years of age [1]. The clinical evolution is heterogeneous as some patients present with remittent-recurrent disease with variable delay between recurrences, while some suffer from chronic disease, with persistent activity over years [1]. For patients with frequent recurrences or chronic disease, topical corticosteroid and immunosuppressants such as cyclosporine A (CSA) and tacrolimus seem a good option, suggesting involvement of underlying immunologic mechanisms [8–10]. However, there are currently no validated criteria to clearly define group of patients according to the clinical course and severity. Additionally, there are no published controlled clinical trials and, consequently, no clear therapeutic guidelines.

The assessment of quality of life (QoL) of patients with TSPK has not been reported in the literature even though recurrent episodes of keratitis could have a significant impact both professionally and socially [11].

Patient support groups formed through online social networks offer a new opportunity for patients with rare diseases to exchange information worldwide on their condition. Facebook is the most popular online social network. This online social media platform was founded in 2004, has more than 2.4 billion active monthly users [12].

Physicians and scientists can therefore take the opportunity to reach out to patients worldwide through online patient support groups, to collect data on rare diseases, across the global demographic [13–17] but, to the best of our knowledge, there are no such studies on ophthalmic diseases. The present study evaluated the burden of disease of TSPK, including clinical journey of TSPK patients and the impact on patient quality of life (QoL) using an online survey targeting a TSPK-specific patient support group on Facebook.

## Patients and methods

This non-interventional study involved the evaluation of results from an online survey, which was sent to a Facebook support group of patients with TSPK [18]. The questionnaire was posted to the online group, and available for three months. If the patients were children or minors, a parent could fill out the form. Data collection was completely anonymous and performed using a French not-for-profit network promoting digital freedom, respectful of data privacy (Framaforms) [19]. This non-interventional study was conducted in compliance with good clinical practice and the tenets of the Declaration of Helsinki. Institutional Review Board/Ethics Committee approval was obtained. The study protocol was reviewed by the Clinical Research Department of Paris Saclay-University and approved by a French research ethics committee (Comité d’éthique de la Recherche—CER—Paris Descartes, N° 2018-59 ROUSSEAU) and declared to the French committee for freedom with informatics data.

The questionnaire was divided in two parts. The first part used 29 questions to gather data on demographics, the clinical journey of patients including diagnostic modalities, symptoms, duration and frequency of recurrent episodes, efficacy and tolerance of treatments. The full questionnaire is available online (Additional file 1). The second part focused on the quality of life of patients affected by TSPK, using the Ocular Surface Disease Quality of Life (OSD-QoL) questionnaire which has been created to evaluate quality of life of patients suffering from ocular surface diseases [20]. The OSD-QoL questionnaire includes 28 questions divided into seven dimensions: daily activities, difficulties with work and handicap, halting the use of make-up, acknowledgement of the disease, acceptance of the disease, fear of the future and emotional well-being. Responses were pooled into three groups: “severe” impact for the most negative answers to the items, “moderate” impact grouping the intermediate answers and “mild” impact for the most positive answers. A total OSD-QoL score was calculated, and ranges from 100 (no impact) to 0 (maximum impact). Patient anonymity was maintained throughout data collection and statistical analysis phases of this study. Descriptive statistical analysis was performed with Excel for Windows (Microsoft Office 365-2019; Microsoft Corp., Redmond, WA, USA).

## Results

### Demographics

Out of 595 members registered on the Facebook TSPK patient support group at the time of the survey, 72 patients (12%) completed the questionnaire from November 2018 to January 2019. There were 58 (81%) females and 14 (19%) males, aged 30 ± 13 years (range 5–59 years). The majority of patients were Caucasian (75%) and living in North America (64%) (Additional file 2: Table S1).

### Clinical journey of patients with TSPK

**Fig. 1:**
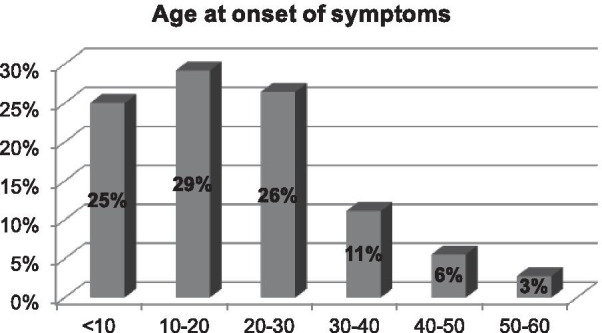
Patient-reported age at onset of symptoms of Thygeson’s punctate superficial keratitis

**Fig. 2:**
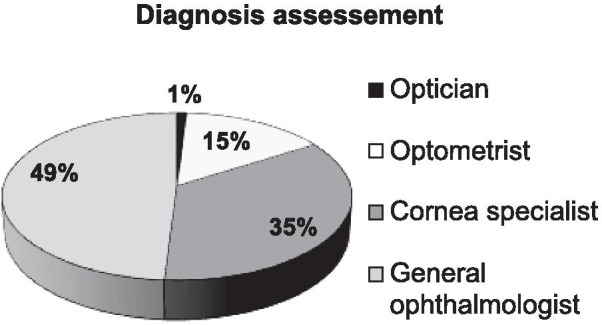
Diagnosis and assessment of patients with Thygeson’s punctate superficial keratitis who responded to an online survey

**Fig. 3:**
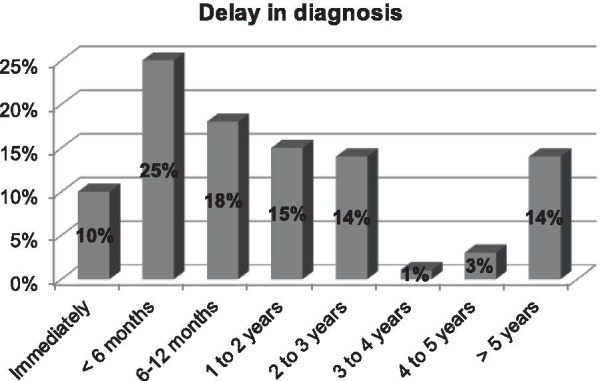
Patient-reported delay in diagnosis of Thygeson’s punctate superficial keratitis among respondents to an online survey

**Fig. 4:**
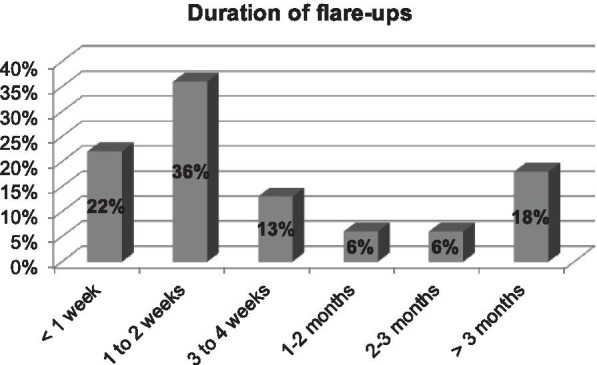
Patient-reported duration of recurrent episodes of Thygeson’s punctate superficial keratitis among respondents to an online survey

**Fig. 5:**
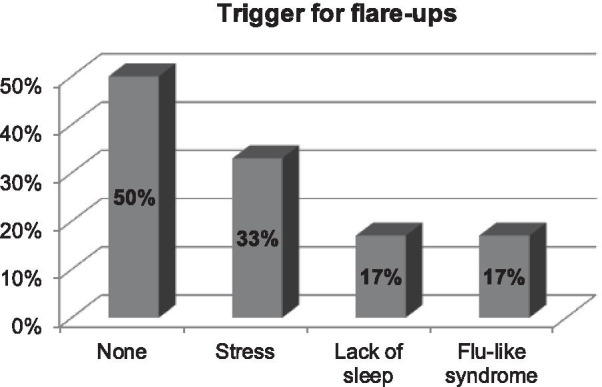
Patient-reported triggers for recurrent episodes of Thygeson’s punctate superficial keratitis among respondents to an online survey

Eighty percent of the patients initially developed symptoms before the age of 30 years and 25% before the age of 10 years (Fig. 1). An ophthalmologist diagnosed TSPK in the majority of cases (Fig. 2). Forty-seven percent of patients were diagnosed more than a year after onset of symptoms (Fig. 3). The disease was usually bilateral (68%), with 60% reported more than 5 recurrent episodes yearly. The duration for recurrences was less than 2 weeks in 58% of patients, while it was more than 3 months (i.e. chronic) in 18% (Fig. 4). Thirty percent of the patients identified stress as a trigger for recurrences, while recurrences were associated with lack of sleep or flu-like syndrome in 17% of patients respectively (Fig. 5).

### Association with autoimmune and inflammatory diseases

Nine patients (13%) declared a history of inflammatory or autoimmune disease such as psoriasis, systemic lupus erythematosus, Sjögren’s syndrome, Hashimoto's thyroiditis or Crohn's disease. None of the patients reported myasthenia gravis, coeliac disease or type 1 diabetes mellitus.

### Efficiency and tolerance of treatments

Steroid eyedrops were self-reported as efficacious by 97% of patients and very efficient in 44% of patients. Thirty percent of patients stated a cumulative duration of topical ophthalmic steroid therapy for more than 5 years*.* Therapeutic contact lenses had been used by 36% of patients and considered efficacious by 68% of these patients whereas lubricants were considered not efficacious by 48% of patients (Additional file 2: Table S2 and S3).

Cyclosporine eyedrops were used by 40% of patients and perceived as somewhat efficacious by 72% of these patients and very efficacious by 17% and were well tolerated by 48%. Cyclosporine eyedrops were used by 26% of patients with less than 5 recurrent episodes per year and 47% of patients with more than 5 recurrent episodes per year. Among patients with chronic disease (episodes lasting > 3 months, N = 13), 7 patients were treated with immunomodulatory eyedrops (cyclosporine, N = 5; tacrolimus, N = 1; lifitegrast, N = 1) and 6/7 patients reported moderate to complete efficiency.

### Impact on the patient quality of life

Overall, the average OSD-QoL score was 55.8 ± 17.5. The impact on daily life activities was moderate for 53% of patients and severe for 22% of patients. Impact on professional and physical activities was moderate to severe for 66% of patients and 38% had to reduce their professional activity. Seventy-eight percent and 54% of patients reported that their eye problems were not acknowledged or barely acknowledged by other people or clinicians respectively. Sixty-eight percent of patients feared never being cured or have greater impairment over time. 80% reported being deeply saddened by their eye condition (Additional file 2: Table S4).

## Discussion

To our knowledge, this is the first study using Facebook to report data on an ophthalmological condition. TSPK is a rare disease and a Facebook support group where large numbers of patients from diverse geographical and ethnic backgrounds can meet “virtually” is an interesting option to collect data on natural history, therapeutic practice patterns and evaluate the impact of disease on patient QoL, thus evaluating the clinical burden of the disease. The use of a social network for ophthalmic research can be an efficient, cost-effective and allow rapid collection of data on patients suffering from rare diseases worldwide.

In our study, patients were mainly Caucasian females younger than 30 years. This finding is consistent with previous epidemiological studies of TSPK, that report a greater preponderance in females than males, with most patients experiencing initial symptoms before 30 years of age [1, 4]. Notably, a diagnostic delay of more than 1 year was noted in nearly half of the patients, highlighting the need for more information on this disease within the eye healthcare community. The age at onset of TSPK-related symptoms was comparable to previously published data, although we had proportionally less patients with an onset after 30 [1]. Although TSPK was originally described as a bilateral process, 32% of patients reported unilateral TSPK, versus 20% in a previous study [1]. However, Nagra et al. [1] reported marked asymmetry in almost half of the patients. Nearly two-thirds of patients reported frequent recurrences (more than 5 episodes per year) and one-fifth reported long lasting episodes that can be considered a chronic disease. Clinically, it is imperative to detect these specific categories of patients as soon as possible to avoid repeated and potentially deleterious corticosteroid usage and to start topical immunomodulatory treatments. Half of our patients reported triggers for recurrent episodes. To our knowledge, this finding had not been studied, even if endogenous and exogenous triggers are common in inflammatory diseases [21].

Our study does not provide information about the duration of disease, as long as most of responders had active disease when they responded. Although sometimes considered as a self-limited disease, TSPK has a long-term course in a significant subset of patients. In their study, Nagra et al. [1] reported a mean duration of 11.1 years, and one patient with active disease 26 years after onset.

The pathophysiology of TSPK remains ambiguous. A viral infection or a virus-induced immune reaction have been implicated [1], based on some clinical similarities with viral keratoconjunctivitis, electron microscopy features of epithelial cells [5], and the favorable response of some patients to trifluridine eyedrops [6]. However, previous studies failed to find genetic materials of common ocular viral pathogens such as varicella zoster virus (VZV), herpes simplex virus (HSV), or adenovirus in samples of affected patients [4, 7]. Alternately, TSPK has been associated with specific immunophenotypic backgrounds such as human leukocyte antigen-DR3 (HLA-DR3), a class-II MHC molecule associated with multiple autoimmune disorders such as diabetes mellitus, gluten enteropathy, systemic lupus erythematosus, Addison's and Sjögren’s syndromes [5]. Among associated autoimmune disorders (14%), one patient had SLE, which is frequently associated with histocompatibility antigen HLA-DR3 [22]. However, none had diabetes mellitus type 1 or celiac disease which are the most common diseases associated with this HLA antigen [23, 24].

Limited data are available in the literature regarding treatment modalities for TSPK. In the current study, therapeutic contact lenses were commonly used in patients with frequently recurrent and/or chronic diseases and perceived as efficacious by more than half of the patients. As expected, topical steroids were considered efficacious in most cases. Unfortunately, nearly one-third of patients reported a long cumulative duration of steroid treatments. Corticosteroid eyedrops are known to be consistently efficacious in TSPK [1], but recurrences occur as they are tapered. Additionally, some have speculated that corticosteroids may increase the duration of disease [1]. Hence, some studies have proposed topical immunomodulatory agents, such as CSA or tacrolimus eyedrops as steroid sparing agents in these patients [9, 10, 25, 26]. Reinhard and Sundmacher’s retrospective study of 52 patients treated with 2% CSA eyedrops reported an efficacy of 71% in adults and 40% in children [9]. However, the majority of patients experienced recurrences when CSA was interrupted and 30% of patients had no recurrences after a 6-month course of treatment [9]. Shoughy and Tabbara reported good outcomes and a rapid response to treatment (72 h) on a series of 10 TSPK patients treated with 0.02% tacrolimus eyedrops [10]. These promising outcomes highlight the need for a controlled study on the treatments for TSPK to determine the best indications, duration and dosage of treatments [9, 10]. Currently, therapeutic strategy is based on an individual ophthalmologist’s experience. As recently highlighted by Ledford, social media, including Facebook may exert a significant influence on the inception and design of clinical trials [27].

The OSD-QoL questionnaire allowed us to assess the impact of TSPK on patient-related QoL. Evaluating QoL is imperative for managing ocular surface disease [28]. To our knowledge, our study is the first to evaluate the QoL of patients with TSPK. The OSD-QoL questionnaire was appropriate in this setting because it calculates a validated score for patients with ocular surface diseases, the calculation itself is simple, the questionnaire is not too long and it was easily adapted for an online survey. The OSD-QoL has been used for other ocular surface diseases such as dry eye disease and herpes simplex keratitis [11, 29]. In our study, the OSD-QoL score of 55.8 ± 17.5 highlights the severe impairment of TSPK on the patient-related QoL. This score indicates that QoL is at least as severely impacted as the QoL among patients suffering from herpes keratitis (65.4 ± 2.9) [11], but with a greater variability. Detailed analysis shows that similar to other ocular surface diseases, patients lack information about the disease, and their condition is poorly understood by their family, friends and their healthcare providers. Similar to dry eye patients, this chronic OSD may cause anxiety / depression, as nearly 80% of patients were “deeply saddened” by their ophthalmic condition in our study [30, 31].

Our study has some limitations inherent to its methodology. Of most concern, severe patients and/or those with a greater impact of TSPK on QoL are likely over-represented among responders, as is often the case for surveys based on voluntary and unremunerated participation. Because of this response bias, results may not represent the complete spectrum of disease. However, it is likely that responders would be those for whom there is an unmet therapeutic need. These patients expressed an overall negative perception of their condition due to its chronic nature, impacting their QoL, and were prescribed potentially harmful treatments in the absence of guidelines. Reporting bias is also inherent to our study since the diagnosis of TSPK could not be ascertained although it was reportedly diagnosed by an ophthalmologist for most patients. Additionally, our results may be limited by a non-response bias (12% of responders despite 3 reminders during the study period) [32]. Response rates to online surveys are usually low, unless a reward is proposed [33]. The response rate are higher for shorter surveys [34]. In our study, the questionnaire was comprised of 57 questions and required more than 10 min to complete, highlighting the challenging balance between duration and the volume (and detail) of data collected. In the context of social networks, response rate also depends on the usage frequency which may vary considerably among users. In the current study, the majority of patients were North American and Caucasian. This could be a selection bias as the survey was in English and Facebook has a higher penetration rate in North America (69%) than Southern Asia (27%) or Western Africa (14%) [35]. This may limit the generalizability of data collected from online social networks. Additionally, ethics issues may arise regarding the use of social network data, particularly regarding anonymity and confidentiality. To mitigate these concerns, we ensured complete anonymity of data, adhered to strict patient confidentiality guidelines, and received research ethics board approval.

## Conclusions

In summary, we collected important and new data on the patient’s clinical journey and the burden of disease of TSPK, a rare and still misunderstood ocular disease. The majority of participants suffers from frequent recurrences and/or chronic disease is common and results in a severe impact on QoL. Additionally, there is frequent off-label use of topical immunomodulatory eyedrops, suggesting the still unmet need for controlled studies to standardize therapeutic protocols. This study also highlights the potential use of social networks for research in rare ophthalmic diseases for rapid collection of data from a diverse sample of patients and thus raises relevant questions about their real needs.

## Supplementary Information

**Additional file 1:** Online questionnaire for Thygeson superficial puncate keratitis patients.

**Additional file 2: Supplementary table 1:** Patients demographics. **Supplemetary table 2:** Patient-reported treatment efficacy. **Supplementary table 3:** Patient-reported treatment tolerance. **Supplementary table 4:** Distribution of patients according to their answers to the OSD-QoL items.

## Data Availability

All data generated or analysed during this study are included in this published article and its supplementary information files.
