# Supplementary material for: Using pre-existing social networks to determine the burden of disease and real-life needs in rare diseases: the example of Thygeson's superficial punctate keratitis
Source: Orphanet J Rare Dis. 2021 Jan 30;16:55. doi: 10.1186/s13023-021-01707-6 (PMC7847580; doi:10.1186/s13023-021-01707-6)
Supplement: Supplementary file 1 — Additional file 1: Online questionnaire for Thygeson superficial puncate keratitis patients. [file 13023_2021_1707_MOESM1_ESM.docx]

TSPK Questionnaire

Thank you for agreeing to take part in this important survey. This questionnaire will help us find out more about Thygeson´s superficial punctate keratitis (TSPK) and how it affects your daily life.

Please fill in this questionnaire somewhere quiet and preferably on your own. Take all the time you need. If you are not sure how to reply, choose the answer that is closest to your situation.

There is no "right" or "wrong" answers.

In case of children or minors affected by the disease, the form can be filled by either parents.

The survey should only take 5 to 10 minutes, and your responses are completely anonymous. Data will be stored and analyzed in France and will not be available for external use.

We really appreciate your input!

1. I agree for the data I provide to be used in this research study. As a reminder, data will be stored and analyzed in France and will not be available for external use. *

- Yes
- No

1. I am filling this questionnaire for: *

- Myself
- Someone else

1. If you are not filling the questionnaire for yourself, who are you filling it for?

|  |
| --- |

Questions below refer only to the person suffering from TSPK.

1. What is your gender? *

- Female
- Male

1. How old are you? *

|  |
| --- |

1. At what age did you experience the first symptoms? *

- Under 10
- Between 10 and 20
- Between 20 and 30
- Between 30 and 40
- Between 40 and 50
- Between 50 and 60
- Between 60 and 70
- Between 70 and 80
- Above 80

1. How long did it take to make the diagnosis of TSPK? *

- Immediately
- Less than 6 months
- Between 6 months and a year
- Between 1 and 2 years
- Between 2 and 3 years
- Between 3 and 4 years
- Between 4 and 5 years
- More than 5 years

1. Do you have a family history of TSPK? (a first degree relative = mother, father, sibling, children) *

- Yes
- No

1. If yes, who?

|  |
| --- |

1. Do you have myasthenia gravis? *

- Yes
- No

1. Do you have type 1 diabetes? *

- Yes
- No

1. Do you have coeliac disease? *

- Yes
- No

1. Do you have an autoimmune disease? *

- Yes
- No

1. If yes, which one?

|  |
| --- |

1. Do you have any other disease not cited above ? If yes, which one ?

|  |
| --- |

1. Do you take any drugs or medication? If yes, which one ?

|  |
| --- |

1. Did you take any drugs or medication within 2 weeks of your first flare up? If yes, which one?

|  |
| --- |

1. Did you identify any triggers for flare-ups? If yes, which one ?

|  |
| --- |

1. On average over the year, how many flare ups do you have? *

- 1
- 2
- 3
- 4
- 5
- More than 5

1. How long does your flare up last on average? *

- Less than a week
- Between 1 week and 2 weeks
- Between 3 weeks and 4 weeks
- Between 1 month and 2 months
- Between 2 months and 3 months
- More than 3 months

1. Does TSPK affect both eyes at the same time? *

- Yes
- No

1. Where do you live? *

- Africa
- Central Asia
- Eastern Asia
- North America
- South America
- Eastern Europe
- Western Europe
- Middle East
- Oceania

1. Where did you spend your childhood? *

- Africa
- Central Asia
- Eastern Asia
- North America
- South America
- Eastern Europe
- Western Europe
- Middle East
- Oceania

1. To which population or ethnic group do you belong? This data is analyzed in order to understand if the ethnic background can predispose to this disease.

- Asian
- African (without Maghreb)/Afroamerican
- Caucasian
- Hispanic
- Indian
- Maghrebian or from Arabian Peninsula
- Other

1. Do you smoke tobacco? *

- Yes
- No, I have never smoked.
- No, I stopped smoking.

1. What type of environment do you spend most of your time in? *

- Outdoor
- Indoor


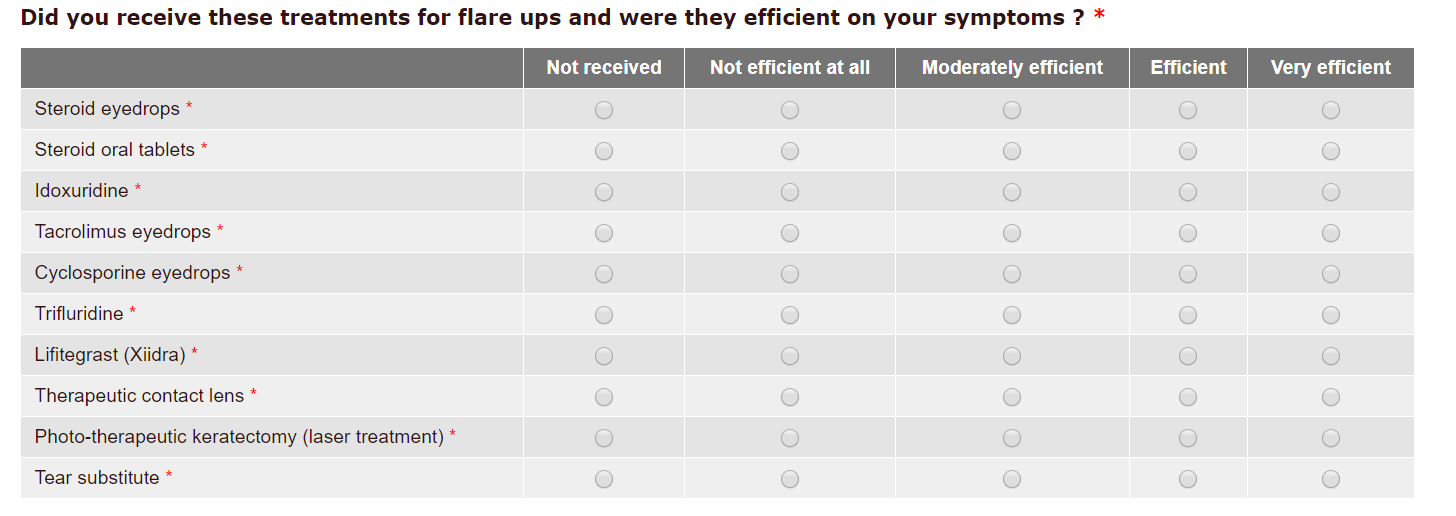


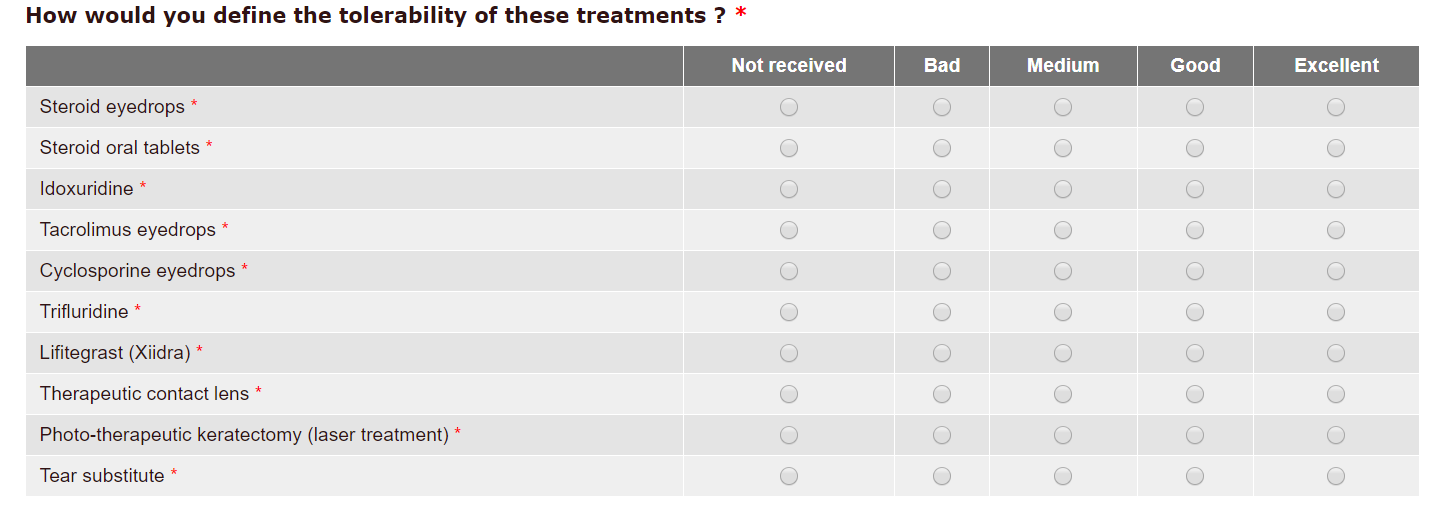


1. Did you receive any other treatments for flare ups?

- Yes
- No

1. If yes, which one ?

|  |
| --- |

1. Do you have steroid drops on a daily basis? If yes, for how long? *

- I don't have steroid drops on a daily basis
- Less than 5 years
- 5 to 10 years
- More than 10 years

1. How long did you take steroid drops (cumulative dose on a lifetime)? *

- I never had steroid drops
- Less than 5 years
- 5 to 10 years
- More than 10 years

1. Do you have ocular hypertension? *

- Yes
- No

1. Do you receive eye drops to reduce your eye pressure? *

- Yes
- No

**Ocular Surface Disease Questionnaire**

1. Because of your eye problems, how often do you have to spend more time carrying out your daily activities (looking after your house, doing your housework, DIY, etc.)? *

- Always
- Most of the time
- Sometimes
- Rarely
- Never

1. Because of your eye problems, how often do you get tired carrying out your daily activities? *

- Always
- Most of the time
- Sometimes
- Rarely
- Never

1. Because of your eye problems, to what extent do you find it difficult to carry out household tasks (minor DIY, housework, cooking, etc.)? *

- A great deal
- A lot
- Somewhat
- A little
- Not at all

1. Because of your eye problems, to what extent do you find it difficult (even with your glasses) to read books, magazines or newspapers? *

- A great deal
- A lot
- Somewhat
- A little
- Not at all
- This question does not apply to me

1. Because of your eye problems, to what extent do you find it difficult (even with your glasses) to watch television? *

- A great deal
- A lot
- Somewhat
- A little
- Not at all
- This question does not apply to me

1. Because of your eye problems, to what extent have you had to give up work? *

- Not at all
- Partially
- Completely
- This question does not apply to me

1. Because of your eye problems, to what extent have you had problems in your career? *

- A great deal
- A lot
- Somewhat
- A little
- Not at all
- This question does not apply to me

1. Because of your eye problems, to what extent do you find it difficult (even with your glasses) to work on a computer? *

- A great deal
- A lot
- Somewhat
- A little
- Not at all
- This question does not apply to me

1. Because of your eye problems, to what extent do you find it hard (even with your glasses) to carry out your work normally? *

- A great deal
- A lot
- Somewhat
- A little
- Not at all
- This question does not apply to me

1. Because of your eye problems, to what extent do you find it difficult carrying out sporting or physical activities? *

- A great deal
- A lot
- Somewhat
- A little
- Not at all
- This question does not apply to me

1. Because of your eye problems, to what extent have you had to stop using make-up? *

- Completely
- Almost completely
- More or less
- A little
- Not at all
- This question does not apply to me

1. Do you feel that people take your eye problems into account? *

- Not at all
- A little
- Somewhat
- A lot
- A great deal

1. Do you feel that doctors take your eye problems into account? *

- Not at all
- A little
- Somewhat
- A lot
- A great deal

1. To what extent do you avoid talking about your eye problems to your friends and family? *

- Not at all
- A little
- Somewhat
- A lot
- A great deal

1. How afraid are you that your eye problems will never be cured? *

- Not at all
- A little
- Somewhat
- Very
- Extremely

1. How afraid are you that your eye problems will get worse? *

- Not at all
- A little
- Somewhat
- Very
- Extremely

1. Do you ever think about the possibility of losing your sight? *

- Always
- Most of the time
- Sometimes
- Rarely
- Never

1. Do you consider your eye problems as serious? *

- Not at all
- A little
- Somewhat
- Very
- Extremely

1. How do you feel about your eye problems? Please choose the expression that corresponds best to how you feel. *


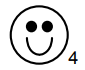

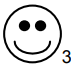

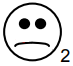

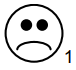

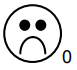


1. Because of my eye problems, I realize that nothing makes me happy anymore. *

- Completely true for me
- Fairly true for me
- Not really true for me
- Not at all true for me

1. Because of my eye problems, I have trouble coping with things. *

- Completely true for me
- Fairly true for me
- Not really true for me
- Not at all true for me

1. Because of my eye problems, I feel more and more discouraged. *

- Completely true for me
- Fairly true for me
- Not really true for me
- Not at all true for me

1. Because of my eye problems, I wake up in the morning feeling depressed. *

- Completely true for me
- Fairly true for me
- Not really true for me
- Not at all true for me

1. Because of my eye problems, I have worries that keep me awake. *

- Completely true for me
- Fairly true for me
- Not really true for me
- Not at all true for me

1. Because of my eye problems, I find the days are never-ending. *

- Completely true for me
- Fairly true for me
- Not really true for me
- Not at all true for me

1. Because of my eye problems, I feel nervous and/or tense. *

- Completely true for me
- Fairly true for me
- Not really true for me
- Not at all true for me

1. Because of my eye problems, I get angry easily. *

- Completely true for me
- Fairly true for me
- Not really true for me
- Not at all true for me

1. Because of my eye problems, I find life not worth living. *

- Completely true for me
- Fairly true for me
- Not really true for me
- Not at all true for me
