# Supplementary material for: Using pre-existing social networks to determine the burden of disease and real-life needs in rare diseases: the example of Thygeson's superficial punctate keratitis
Source: Orphanet J Rare Dis. 2021 Jan 30;16:55. doi: 10.1186/s13023-021-01707-6 (PMC7847580; doi:10.1186/s13023-021-01707-6)
Supplement: Supplementary file 2 — Additional file 2: Supplementary table 1: Patients demographics. Supplemetary table 2: Patient-reported treatment efficacy. Supplementary table 3: Patient-reported treatment tolerance. Supplementary table 4: Distribution of patients according to their answers to the OSD-QoL items. [file 13023_2021_1707_MOESM2_ESM.docx]

**Supplementary tables:**

| Screened patients | 595 |
| --- | --- |
| Included patients | 72 (12%) |
| Female | 58 (81%) |
| Males | 14 (19%) |
| Mean age (years) | 30±13 |
| **Current residence** | |
| Eastern Asia | 3% |
| Eastern Europe | 1% |
| North America | 64% |
| Oceania | 11% |
| South America | 3% |
| Western Europe | 18% |
| **Childhood residence** | |
| Central Asia | 3% |
| Eastern Asia | 1% |
| Eastern Europe | 3% |
| North America | 63% |
| Oceania | 8% |
| South America | 4% |
| Western Europe | 18% |
| **Ethnicity** | |
| African (excluding Maghreb) or Afro-american | 3% |
| Asian | 7% |
| Caucasian | 75% |
| Hispanic | 6% |
| Indian | 1% |
| Maghrebian or from Arabian Peninsula | 1% |
| Other | 7% |

**Supplementary table 1:** patients demographics.

| Treatments | Not received | Not efficient at all | Moderately efficient | Efficient | Very efficient |
| --- | --- | --- | --- | --- | --- |
| Steroid eyedrops | 2 (3%) | 2 (3%) | 19 (27%) | 18 (26%) | 31 (44%) |
| Steroid oral tablets | 71 (99%) | 1 (100%) | 0 | 0 | 0 |
| Idoxuridine | 72 (100%) | 0 | 0 | 0 | 0 |
| Tacrolimus eyedrops | 70 (98%) | 1 (50%) | 1 (50%) | 0 | 0 |
| Cyclosporine eyedrops | 43 (60%) | 8 (28%) | 14 (48%) | 2 (7%) | 5 (17%) |
| Trifluridine | 68 (94%) | 2 (50%) | 0 | 2 (50%) | 0 |
| Lifitegrast | 67 (93%) | 2 (40%) | 1 (20%) | 2 (40%) | 0 |
| Therapeutic contact lens | 53 (74%) | 6 (31.5%) | 6 (31.5%) | 3 (16%) | 4 (21%) |
| Phototherapeutic keratectomy | 68 (94%) | 1 (25%) | 2 (50%) | 1 (25%) | 0 |
| Tear substitute | 24 (33%) | 23 (48%) | 18 (37.5%) | 6 (12.5%) | 1 (2%) |

**Supplementary table 2:** Patient-reported treatment efficacy.

| Treatments | Not received | Bad | Medium | Good | Excellent |
| --- | --- | --- | --- | --- | --- |
| Steroid eyedrops | 2 (3%) | 5 (7%) | 20 (28.5%) | 23 (33%) | 22 (31.5%) |
| Steroid oral tablets | 71 (99%) | 0 | 0 | 0 | 1 (100%) |
| Idoxuridine | 72 (100%) | 0 | 0 | 0 | 0 |
| Tacrolimus eyedrops | 70 (98%) | 0 | 2 (100%) | 0 | 0 |
| Cyclosporine eyedrops | 43 (60%) | 6 (21%) | 9 (31%) | 7 (24%) | 7 (24%) |
| Trifluridine | 68 (94%) | 1 (25%) | 2 (50%) | 1 (25%) | 0 |
| Lifitegrast | 67 (93%) | 1 (20%) | 1 (20%) | 2 (40%) | 1 (20%) |
| Therapeutic contact lens | 53 (74%) | 3 (16%) | 6 (31%) | 7 (37%) | 3 (16%) |
| Photo-therapeutic keratectomy | 68 (94%) | 2 (50%) | 2 (50%) | 0 | 0 |
| Tear substitute | 24 (33%) | 5 (10.5%) | 12 (25%) | 14 (29%) | 17 (35.5%) |

**Supplementary table 3:** Patient-reported treatment tolerance.

| Dimensions | Item # and conceptual content | | Patients reporting  severe impact | Patients reporting moderate impact | Patients reporting no or little impact |
| --- | --- | --- | --- | --- | --- |
| Dailies activities | 1  2  3  4  5 | Taking longer over your daily activities  Being tired by doing your daily activities  Having difficulty doing housework  Having difficulty reading  Having difficulty watching television | 16 (22%)  28 (39%)  20 (28%)  33 (47%)  33 (46%) | 30 (42%)  26 (36%)  26 (36%)  18 (25%)  17 (24%) | 26 (36%)  18 (25%)  26 (36%)  20 (28%)  21 (30%) |
| Difficulties  with work  and handicap | 6  7  8  9  10 | Stopping working due to eye problem  Hindrance of your professional career  Having difficulty using a computer at work  Having difficulty at work  Having difficulty with physical activity | 3 (5%)  21 (36%)  34 (49%)  28 (42%)  18 (26%) | 27 (45%)  14 (24%)  17 (25%)  11 (17%)  19 (27%) | 30 (50%)  23 (40%)  18 (26%)  27 (41%)  33 (47%) |
| Giving up  make-up | 11 | Giving up using make-up | 26 (51%) | 5 (10%) | 20 (39%) |
| Acknowledgement of the disease | 12  13 | Eye problems acknowledged by people  Eye problems acknowledged by clinicians | 55 (76%)  39 (54%) | 12 (17%)  23 (32%) | 5 (7%)  10 (14%) |
| Acceptance  of the disease | 14 | Avoiding speaking about your eye problems | 16 (22%) | 23 (32%) | 33 (46%) |
| Fear for the future | 15  16  17  18  19 | Being afraid of never being cured  Being afraid of becoming more impaired  Being afraid of going blind  Judging your eye problems as serious  How do you feel ? | 48 (67%)  47 (65%)  18 (25%)  47 (65%)  57 (79%) | 15 (21%)  15 (21%)  32 (44%)  16 (22%)  13 (18%) | 9 (12%)  10 (14%)  22 (31%)  9 (13%)  2 (3%) |
| Emotionnal well-being | 20  21  22  23  24  25  26  27  28 | No longer enjoying anything (experience pleasure)  Having difficulty coping with life  Feeling more and more discouraged  Waking up depressed in the morning  Having difficulty getting to sleep due to worries  Thinking working days are endless  Feeling nervous or anxious  Getting angry easily  Thinking that life is not worth living | 2 (3%)  3 (4%)  1 (1%)  3 (4%)  3 (4%)  3 (4%)  6 (8%)  10 (14%)  2 (3%) | 7 (10%)  19 (26%)  23 (32%)  9 (13%)  7 (10%)  15 (21%)  17 (24%)  9 (12%)  1 (1%) | 63 (87%)  50 (69%)  48 (67%)  60 (83%)  62 (86%)  54 (75%)  49 (68%)  53 (74%)  69 (96%) |

**Supplementary table 4:** Distribution of patients according to their answers to the OSD-QoL items
